# Segmentation error in spectral domain optical coherence tomography measures of the retinal nerve fibre layer thickness in idiopathic intracranial hypertension

**DOI:** 10.1186/s12886-017-0652-7

**Published:** 2018-01-04

**Authors:** Anuriti Aojula, Susan P Mollan, John Horsburgh, Andreas Yiangou, Kiera A Markey, James L Mitchell, William J Scotton, Pearse A Keane, Alexandra J Sinclair

**Affiliations:** 1 Institute of Metabolism and Systems Research, University of Birmingham, B15 2TT, Birmingham, UK; 2 Centre for Endocrinology, Diabetes and Metabolism, Birmingham Health Partners, B15 2TH, Birmingham, UK; 3 Birmingham Neuro-Ophthalmology Unit, University Hospitals Birmingham NHS Foundation Trust, Queen Elizabeth Hospital, Edgbaston, B15 2TH, Birmingham, UK; 4 NIHR Biomedical Research Centre for Ophthalmology, Moorfields Eye Hospital NHS Foundation Trust and UCL Institute of Ophthalmology, London, UK; 5 Department of Neurology, University Hospitals Birmingham NHS Foundation Trust, B15 2TH, Birmingham, UK

**Keywords:** Papilloedema, Idiopathic intracranial hypertension, Pseudotumour Cerebri, Optical coherence tomography, Retinal nerve fibre layer, Artefact, Imaging, Monitoring

## Abstract

**Background:**

Optical Coherence Tomography (OCT) imaging is being increasingly used in clinical practice for the monitoring of papilloedema. The aim is to characterise the extent and location of the Retinal Nerve Fibre Layer (RNFL) Thickness automated segmentation error (SegE) by manual refinement, in a cohort of Idiopathic Intracranial Hypertension (IIH) patients with papilloedema and compare this to controls.

**Methods:**

Baseline Spectral Domain OCT (SDOCT) scans from patients with IIH, and controls with no retinal or optic nerve pathology, were examined. The internal limiting membrane and RNFL thickness of the most severely affected eye was examined for SegE and re-segmented. Using ImageJ, the total area of the RNFL thickness was calculated pre and post re-segmentation and the percentage change was determined. The distribution of RNFL thickness error was qualitatively assessed.

**Results:**

Significantly greater SegE (*p* = 0.009) was present in RNFL thickness total area, assessed using ImageJ, in IIH patients (*n* = 46, 5% ± 0–58%) compared to controls (*n* = 14, 1% ± 0–6%). This was particularly evident in moderate to severe optic disc swelling (*n* = 23, 10% ± 0–58%, *p* < 0.001). RNFL thickness was unable to be quantified using SDOCT in patients with severe papilloedema.

**Conclusions:**

SegE remain a concern for clinicians using SDOCT to monitor papilloedema in IIH, particularly in the assessment of eyes with moderate to severe oedema. Systematic assessment and manual refinement of SegE is therefore important to ensure the accuracy in longitudinal monitoring of patients.

**Electronic supplementary material:**

The online version of this article (10.1186/s12886-017-0652-7) contains supplementary material, which is available to authorized users.

## Background

Quantifying papilloedema clinically is subjective and prone to inter–observer variability and inaccuracy during prospective monitoring [1]. Spectral Domain Optical Coherence Tomography (SDOCT) is increasingly used both in the clinical environment and as outcome measures in Idiopathic Intracranial Hypertension (IIH) clinical trials to objectively quantify papilloedema [2]. Commercially available SD-OCT imaging systems, such as the Cirrus HD-OCT (Carl Zeiss Meditec, Dublin, CA) and Spectralis (Heidelberg Engineering, Heidelberg, Germany), have proprietary in-built OCT software logarithms which use the difference in signal intensity between adjacent retinal layers to perform automated segmentation to segment inner and outer retinal boundaries, from which retinal nerve fibre layer thickness (RNFL thickness) measurements can be calculated. Optic disc swelling can be monitored by repeated assessments of the RNFL thickness [2–4]. Autosegmentation has been found to be inaccurate in some retinal pathologies such as neovascular age related macular degeneration and central serous retinopathy [5] and in optic nerve head pathologies such as glaucoma [6]. In papilloedema, where the interface between the retinal layers is disturbed by oedema, errors in autosegmentation have been noted [7–9], with large studies using the Cirrus HD-OCT. The aim of this study was to evaluate the extent and location of the RNFL thickness SegE in an IIH cohort, comparing this to normal controls using the Spectralis SD-OCT.

## Methods

### Subjects

Fifty-two consecutive IIH patients with a mean age of 31 years (standard deviation (SD) 9.4 years) and 14 controls with a mean age of 35.9 years (SD 7.21 years) at University Hospitals Birmingham NHS Foundation Trust (UHB NHS FT), a large tertiary referral centre, were enrolled. All participants were female. The study followed the tenets of the Declaration of Helsinki; informed consent was obtained; and the research was approved as a service evaluation by the UHB NHS FT research and development department. For inclusion, subjects were required to have active disease (papilloedema with at least Frisén grade 1 in one eye) and fulfil the accepted revised diagnostic for IIH [10]. The IIH cohort had a median body mass index (BMI) of 38.7kgm^−2^ (range 24.3–51.3kgm^−2^) and a median baseline lumbar puncture opening pressure (LP OP) of 35.5 cm CSF (25.0–60.0 cm CSF). Using the Modified Frisén Scale [11], two independent reviewers (SPM and JH) stratified anonymised fundal photos according to the degree of papilloedema; 23 were classified as mild disc swelling (Frisén grade 1–2), and 29 with moderate/severe disc swelling (Frisén grade 3–5). Healthy control subjects were recruited only after retinal and/or optic nerve pathology was excluded. Lumbar punctures and BMI indices were not conducted in the control group.

### SDOCT imaging

SDOCT RNFL thickness peripapillary circular scans were acquired from all subjects using Heidelberg Engineering SPECTRALIS HRA + OCT (Heidelberg Engineering, Heidelberg, Germany). Spectralis OCT uses a dual-beam SDOCT, a confocal laser-scanning ophthalmoscope with a wavelength of 870 nm, and an infrared reference image to obtain images of ocular microstructures with an acquisition rate of 40,000 A-scans per second. Sufficient OCT scan quality was considered as a Q score of greater than 12 and the absence of posterior vitreous detachment, fovea malalignment or media opacity secondary to cataract. The Spectralis OCT software allows for automatic segmentation of the upper and lower borders of the RNFL to calculate the average RNFL thickness. Peripapillary RNFL thickness values are divided into 4 quadrants, namely superior, inferior, nasal and temporal. The SD-OCT scans were qualitatively and quantitatively examined for RNFL thickness SegE and analysed with both the Spectralis automated software and then ImageJ Software package (https://imagej.nih.gov/ij/).

### Examination for OCT segmentation error (SegE)

A masked reviewer (AA) assessed anonymised OCT scans for SegE using Heidelberg Eye Explorer software, version 1.9.1. (Heidelberg Engineering, Heidelberg, Germany). For each subject, only one eye was analysed using the baseline OCT scan from the most severely affected eye; this was identified by the highest single point maximum RNFL thickness value (μm). Initially the SegE was qualitatively assessed and the location recorded. Quantitative analysis then involved evaluating the internal limiting membrane and RNFL thickness for the presence of SegE and accordingly using the Heidelberg Eye Explorer software which automatically identifies the layer border and allows for manual correction of the segmentation. Pre- and post re-segmentation, average and maximum height of the RNFL thickness (μm) was recorded in the following areas: global RNFL thickness and the superior, nasal, inferior and temporal retinal quadrant. Finally, pre and post re-segmentation, the RNFL thickness total area was delineated and quantified independently of the Heidelberg Eye Explorer software, using the ImageJ software polygon and analyse area tool, respectively. The percentage change in the total area of the RNFL area was subsequently calculated. Quality assurance was undertaken with a further masked observer (JH) independently examining SegE in a quarter (*n* = 20) of the subjects in the cohort to ensure there was sufficient concordance.

### Statistics

Descriptive statistics were used to compare demographic characteristics by group (IIH and healthy controls). Statistical analysis was performed using SPSS software, version 23.0 (IBM, Armonk, NY). Due to the lack of normal distribution, data were analysed using the Kruskal-Wallis test and tested for significant pairwise comparisons. Values were expressed as the median ±range. A two-tailed Spearman’s correlation test was used to conduct correlation analysis. Values were deemed statistically significant at *p* < 0.05.

## Results

**Fig. 1:**
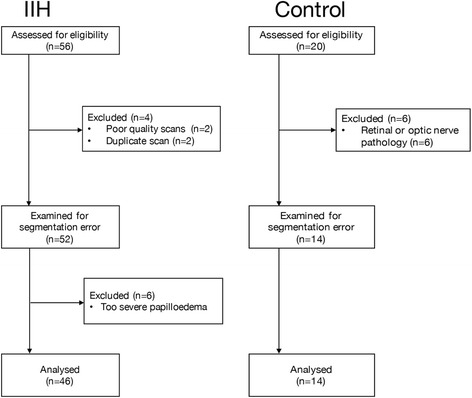
This is showing the consort pathway for inclusion and exclusion of subjects in this study

Of the 52 IIH patients, the scans from 6 patients were excluded as they had such severe papilloedema that the optic nerve head elevation was truncated by the scan image and therefore the height could not be visualised and no further accurate refinement of the RNFL thickness could be performed. Forty-six IIH subjects and 14 controls were therefore included in the quantitative analysis (Fig. 1). The reliability between the two independent raters (AA and JH) was 0.732, with 95% CI (0.232–0.926), *p* < 0.05.

Quantification of the difference between the automated and the manually corrected total RNFL thickness area using ImageJ revealed significantly greater SegE in IIH patients [5% change post segmentation refinement, range = 0–58%] compared to controls [1% change post segmentation refinement, range = 0–6%] *p* = 0.009 (Table 1; Additional file 1: Table S1, S2). This was particularly evident in IIH patients with moderate to severe papilloedema [10% change post segmentation refinement, range = 0–58%, *p* < 0.001]. (Additional file 1: Table S3).

**Table 1:** Qualitative assessment of the distribution of RNFL thickness segmentation error comparing the IIH and control cohorts using median values and ranges

Location	Overall IIH % error (*n* = 46)	Mild IIH % error (*n* = 23)	Moderate-severe IIH % error (*n* = 23)	Control % error (*n* = 14)	p overall	p moderate-severe
Using ImageJ						
Total area of RNFL	5 (0–58)	2 (0–16)	10 (0–58)	1 (0–6)	0.009^a^	<0.001^a^
Using spectalis automated software						
Average						
Overall	4 (0–58)	2 (0–16)	10 (0–58)	2 (0–6)	0.031^a^	0.002^a^
Superior	8 (0–375)	6 (0–115)	11 (0–375)	3 (0–10)	0.007^a^	0.001^a^
Nasal	2 (0–81)	2 (0–30)	1 (0–81)	1 (0–14)	NS	NS
Inferior	4 (0–79)	3 (0–12)	6 (0–79)	2 (0–11)	0.031^a^	0.008^a^
Maximum						
Highest Single Point	5 (0–62)	5 (0–43)	7 (0–62)	4 (0–14)	NS	NS
Superior	7 (0–60)	5 (0–42)	12 (0–60)	3 (0–26)	0.044^a^	0.017^a^
Nasal	6 (0–70)	5 (0–51)	8 (0–70)	7 (0–133)	NS	NS
Inferior	5 (0–96)	3 (0–114)	7 (0–96)	3 (0–17)	NS	0.049^a^
Temporal	3 (0–43)	3 (0–21)	3 (0–43)	1 (0–28)	NS	NS

*NB* values are compared with the control group. There were no significant differences in mild disc swelling for any of the above parameters. *NS* not significant; ^a^indicates statistical significance

**Fig. 2:**
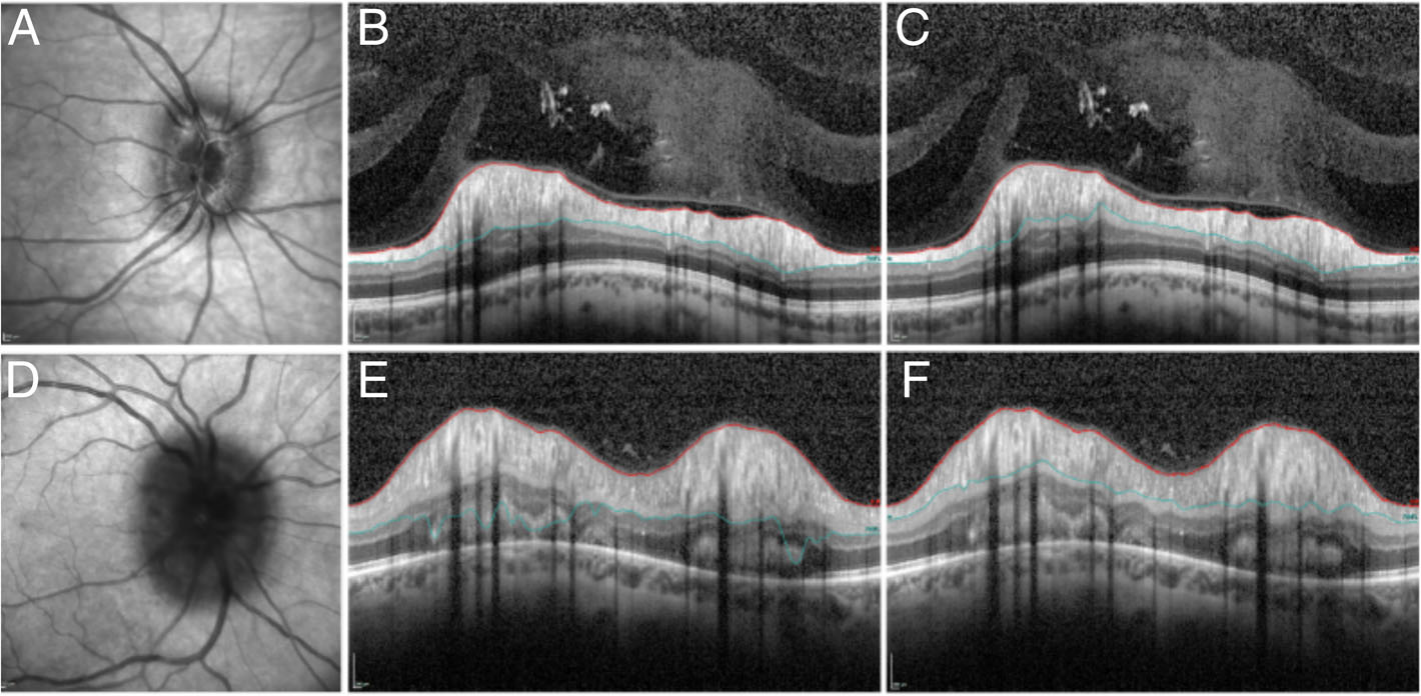
Demonstrates the typical infra-red (IR) images pre and post refinement of the automated segmentation. 1**a**-1**c** is a case of mild disc swelling: 1**a**, IR image of optic nerve head; 1**b**, Cross section of the peripapillary RNFL scan as autosegmented; 1**c**, Cross section of the peripapillary RNFL scan following refinement of the segmentation manually. 1**d**-1**f** is a case of moderate to severe disc swelling: 1**d**, IR image of optic nerve head; 1**e**, Cross section of the peripapillary RNFL scan as autosegmented; 1**f**, Cross section of the peripapillary RNFL scan following refinement of the segmentation manually

**Fig. 3:**
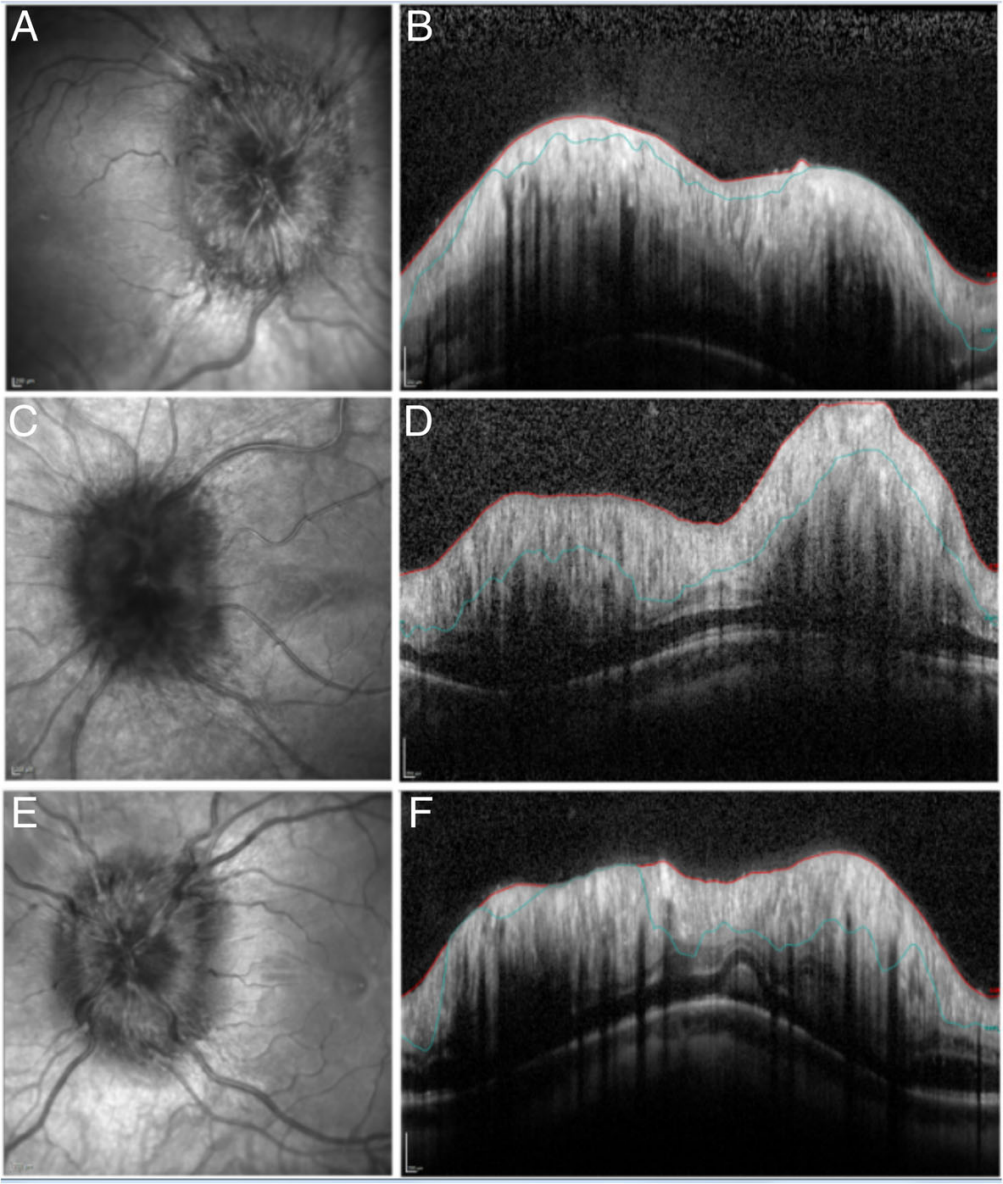
This figure presents the IR image of the optic nerve head and the cross-sectional peripapillary circle scan for three subjects (**ab**, **cd**, **ef**). The figure demonstrates that in moderate to severe optic nerve head swelling the RNFL boundary as delineated by autosegmentation (blue line) is variable and not accurate. The average RNFL thickness values in these cases will be very inaccurate in these patients. In subject CD the height of the elevation of the optic nerve head is truncated by the image width and hence any values obtained from this scan are inaccurate. All the cases presented in these images were not manually segmented and were excluded from the study

The error in automated overall average RNFL thickness values was significantly greater in IIH compared to controls (*p* = 0.031): median176μm (range 76-581 μm) pre re-segmentation versus 159 μm (range 83-391 μm) post re-segmentation in IIH (4% change post re-segmentation, range 0–58%) this was compared to 98 μm (range 63-125 μm) pre segmentation versus 100 μm (range 65-126 μm) post re-segmentation in controls (2% change post re-segmentation, range = 0–6%). IIH patients with moderate to severe papilloedema displayed significantly greater error in the overall average RNFL thickness values compared to those with mild papilloedema [10% change post re-segmentation (range 0–58% in moderate and severe papilloedema, *p* = 0.002)] (Table 1 and Fig. 2). In those with moderate to severe papilloedema the SegE was significantly greater in the superior retinal quadrant [11% change post re-segmentation, range = 0–375%, *p* = 0.001] (Fig. 3).

Qualitative assessment of any error in the RNFL thickness segmentation was more often identified in IIH (any apparent error in 98% of the IIH group, (45/46)) compared to control subjects (error in 79% of patients, (11/14)) (Table 2). It was clearly observed that the magnitude of the SegE was more pronounced in the IIH compared to control subjects. There was no clear pattern between subjects of whether the error was inflation or deflation of their disc height. In IIH patients, the RNFL thickness SegE was predominantly noted in the superior retinal quadrant, but to a lesser degree in the inferior retinal quadrant. In contrast, the control subjects displayed minimal error that had no predominant distribution.

**Table 2:** Qualitative assessment of the distribution of RNFL thickness segmentation error

Error location	IIH (*n* = 46)	Control (*n* = 14)
No error seen	1	3
No predominant distribution	9	8
Superior	23	2
Nasal	3	0
Inferior	10	1
Temporal	0	0

The highest single point of maximum thickness in the RNFL thickness was then assessed and there was no statistically significant difference in RNFL thickness segmentation between the automated and the manually corrected segmentation between IIH and controls. However error in the maximal height in the superior region in those with moderate or severe papilledema did have significant error pre- and post re-segmentation in the superior retinal quadrant: 345 μm (range 139-1007 μm) automated value versus 297 μm (range 178-445 μm) post re-segmentation (12% change post re-segmentation, range = 0–60%, *p* = 0.017), compared to 174 μm (range 144 - 211 μm) automated values and 184 μm (range 150–211 μm) post re-segmentation for the controls (3% change post re-segmentation, range = 0–26%).

## Discussion

OCT imaging is increasingly utilised for quantification and monitoring of papilloedema in IIH in the clinical setting. The largest prospective controlled cohort in papillodema reporting use of OCT in IIH is data from the IIHTT [2]; this study investigated 126 participants and utilised the Cirrus OCT at multiple centres and found that 3-dimensional scanning was less prone to failures of segmentation than 2-dimensional images. There is limited literature on the accuracy of autosegmentation in papilloedema using the Spectralis OCT. Detection of SegE, and manually refining the interfaces could help improve the accuracy of the RNFL thickness values between consecutive tests, and improve the clinical utility of the SD-OCT in longitudinal monitoring of IIH. This study highlights the issue of significant error in the automated RNFL thickness values generated from peripapillary RNFL thickness circle scans using the Spectralis SD-OCT. Like other SD OCT devices, the Spectralis in built algorithms are not specifically designed to autosegment papilloedema and although we identified significant error in the overall average RNFL thickness value with a 4% change following manual re-segmentation, this is much less error than previously reported using time domain OCT platforms [12]. What is yet to be determined is the clinical significance of the magnitude of this error.

The SegE was most apparent in those with moderate to severe papilloedema (10% error) and particularly in the superior retinal quadrant (11% error). The majority of the RNFL thickness error was accounted for by inaccurate automated identification of the lower boarder of the RNFL at the junction with the ganglion cell layer. It is likely that oedema and vessel artefact lead to error in the average RNFL thickness automated values, as postulated by previous authors [9, 13].

In 6% of the cohort, with severe papilloedema, the extreme elevation of the optic nerve head obscured the upper boarder limit of the RNFL and it was therefore not possible to refine the segmentation in these patients due to the truncation of the image. This truncation artefact has been previously reported by other authors [the type 1–8 paper] [9]. It may be less of an issue with newer OCT systems, using swept source technology, that provide a greater depth of imaging (e.g., Topcon DRI OCT-1 Triton has a depth range of 2.6 mm – greater than the 1.9 mm depth range of the Heidelberg system, based on spectral domain technology) [14].

SegE is not the only cause for erroneous RNFL thickness values; a number of factors have been associated with artifacts in OCT scanning including decentration error, refractive error, posterior vitreous detachment artifacts, reduced visual acuity, small pupils, presence of media opacities, advanced stage of glaucoma and dry eyes [9, 15–18] Eye tracking on Spectralis ensures better alignment and is reported to decreases error in malalignment [19, 20].

Manual refinement of segmentation has several limitations which include the time taken to perform this accurately; indeed the accuracy of the manual markings, in which experts invariably disagree on where to draw the margins when the boarders between layers are hazy. In this study we performed an inter-user variability check to ensure that there was sufficient agreement between two masked individuals. The clinical impact of this error has not been evaluated in this study but would be a useful area for future investigation. As the Spectralis SD OCT platform was used in this study our results may not be generalizable to results from other types of SD-OCT machines.

RNFL thickness peripapillary scans are not the only OCT scan modality used to assess papilloedema. Other scanning modalities include volumetric analysis of the optic nerve head and macula; and Bruchs Membrane Opening (BMO) rim analysis. However, the degree of oedema in moderate to severe papilloedema is also known to cause error in these scans due to optical penetration. Future solutions include better depth penetration and a wider scan window to account for the elevation found in disc oedema. Polarization-sensitive OCT, which is not currently commercially available, has the potential to delineate the RNFL boundary better based on pigment differences in the retinal layers and be less prone to SegE [21].

**Fig. 4:**
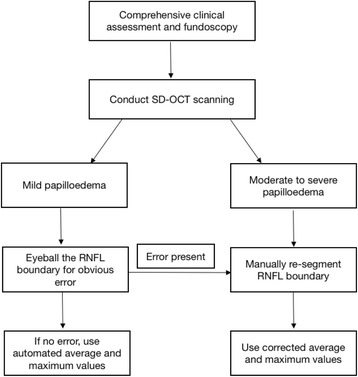
Practical Algorithm for inclusion of SD-OCT RNFL thickness values in the clinical setting

In the setting of virtual IIH clinics where patient’s management may be judged exclusively by objective OCT and Humphrey visual field values; there could be clinical risk in the misinterpretation of the degree of papilledema and its course over time if the SegE is not identified and corrected for at the time of acquisition of the scans [9]. Here we have highlighted the limitations of using automated results from OCT RNFL thickness scans, particularly in those with marked papilloedema. We have developed a suggested paradigm to guide healthcare professionals performing OCT RNFL thickness peripapillary scans in IIH (Fig. 4). Scans should be evaluated for error at the time of acquisition to ensure the accuracy of the data at the time of the clinical visit when management decisions may be being made. However, as demonstrated with the results from the IIHTT [2] we would recommend the use of optic nerve volume scans in the routine clinical assessment of papilloedema.

## Conclusions

Using the Spectralis SD-OCT, SegE in RNFL thickness values were found to be greater in IIH than controls, with the error increasing with the severity of the papilloedema. Imaging was not useful in very severe papilloedema where the image was truncated. This is the largest cohort assessing SegE in IIH, using the Spectralis SD-OCT. Achieving accurate and reproducible image analysis is important in the longitudinal monitoring in IIH, hence recognition of SegE and manual refinement should be understood by technicians and clinicians alike.

## Additional file

Additional file 1: Table S1. IIH Cohort showing absolute median values (range) pre and post segmentation with % change. **Table S2.** Controls showing absolute median values (range) pre and post segmentation with % change. **Table S3.** Moderate to severe IIH showing absolute median values (range) pre and post segmentation with % change. **Table S4.** Mild IIH showing absolute median values (range) pre and post segmentation with % change. (DOCX 35 kb)

## Abbreviations

BMO: Bruchs membrane opening
IIH: Idiopathic intracranial hypertension
OCT: Optical coherence tomography
RNFL: Retinal nerve fibre layer
SDOCT: Spectral domain optical coherence tomography
SegE: Segmentation error
