# Supplementary material for: Segmentation error in spectral domain optical coherence tomography measures of the retinal nerve fibre layer thickness in idiopathic intracranial hypertension
Source: BMC Ophthalmol. 2018 Jan 4;17:257. doi: 10.1186/s12886-017-0652-7 (PMC6389234; doi:10.1186/s12886-017-0652-7)
Supplement: Supplementary file 1 — IIH Cohort showing absolute median values (range) pre and post segmentation with % change. Table S2. Controls showing absolute median values (range) pre and post segmentation with % change. Table S3. Moderate to severe IIH showing absolute median values (range) pre and post segmentation with % change. Table S4. Mild IIH showing absolute median values (range) pre and post segmentation with % change. (DOCX 35 kb) [file 12886_2017_652_MOESM1_ESM.docx]

**Additional file 1**

**Table S1: IIH Cohort showing absolute median values (range) pre and post segmentation with % change.**

| **Location** | **Pre segmentation (µm)** | **Post segmentation**  **(µm)** | **% change** |
| --- | --- | --- | --- |
| **Average** |  |  |  |
| **Overall** | **152(76-581)** | **145(83-391)** | **4(0-58)** |
| **Superior** | **157(73-623)** | **166(91-492)** | **8(0-375)** |
| **Nasal** | **102(45-1045)** | **104(47-364)** | **2(0-81)** |
| **Inferior** | **203(107-504)** | **197(113-402)** | **4(0-79)** |
| **Temporal** | **82(48-381)** | **82(50-307)** | **2(0-44)** |
| **Maximum** |  |  |  |
| **Highest Single Point** | **324 (170-1141)** | **311(171-537)** | **5 (0-62)** |
| **Superior** | **250 (133-1007)** | **246(156-537)** | **7 (0-60)** |
| **Nasal** | **190(75-1141)** | **178(76-497)** | **6(0-60)** |
| **Inferior** | **294(87-984)** | **293(6-496)** | **5(0-96)** |
| **Temporal** | **154(71-661)** | **156(92-400)** | **3(0-43)** |

**Table S2: Controls showing absolute median values (range) pre and post segmentation with % change**

| **Location** | **Pre segmentation (µm)** | **Post segmentation (µm)** | **% change** |
| --- | --- | --- | --- |
| **Average** |  |  |  |
| **Overall** | **97(77-125)** | **101(65-126)** | **2 (0-6)** |
| **Superior** | **116(96-157)** | **118(96-146)** | **3 (0-10)** |
| **Nasal** | **71(40-102)** | **72(41-102)** | **1 (0-14)** |
| **Inferior** | **131(73-169)** | **132(73-170)** | **2 (0-11)** |
| **Temporal** | **72(30-95)** | **72(30-95)** | **1 (0-12)** |
| **Maximum** |  |  |  |
| **Highest Single Point** | **192(161-228)** | **196(171-230)** | **4 (0-14)** |
| **Superior** | **171(144-211)** | **185(150-211)** | **3 (0-26)** |
| **Nasal** | **118(68-194)** | **140(70-194)** | **7 (0-133)** |
| **Inferior** | **192(120-228)** | **196(125-230)** | **3 (0-17)** |
| **Temporal** | **109(79-162)** | **123(80-161)** | **1 (0-28)** |

**Table S3: Moderate to severe IIH showing absolute median values (range) pre and post segmentation with % change**

| **Location** | **Pre segmentation (µm)** | **Post segmentation (µm)** | **% change** |
| --- | --- | --- | --- |
| **Average** |  |  |  |
| **Overall** | **208(150-581)** | **198(120-258)** | **10 (0-58)** |
| **Superior** | **243(73-410)** | **278(76-492)** | **11 (0-375)** |
| **Nasal** | **162(65-1045)** | **151(65-214)** | **1 (0-81)** |
| **Inferior** | **279(154-504)** | **268(143-402)** | **6 (0-79)** |
| **Temporal** | **112(50-381)** | **104(51-307)** | **3 (0-44)** |
| **Maximum** |  |  |  |
| **Highest Single Point** | **431(239-1141)** | **400(234-546)** | **7 (0-62)** |
| **Superior** | **392(139-1007)** | **341(178-546)** | **12 (0-60)** |
| **Nasal** | **284(101-1141)** | **260(153-497)** | **8 (0-70)** |
| **Inferior** | **405(239-984)** | **345(234-496)** | **7 (0-96)** |
| **Temporal** | **263(112-661)** | **233(122-455)** | **3 (0-43)** |

**Table S4: Mild IIH showing absolute median values (range) pre and post segmentation with % change**

| **Location** | **Pre segmentation (µm)** | **Post segmentation**  **(µm)** | **% change** |
| --- | --- | --- | --- |
| **Average** |  |  |  |
| **Overall** | **108(76-208)** | **111(83-180)** | **2 (0-16)** |
| **Superior** | **143(84-290)** | **146(91-279)** | **6 (0-115)** |
| **Nasal** | **81(45-163)** | **84(47-134)** | **2 (0-30)** |
| **Inferior** | **140(107-303)** | **140(113-314)** | **3 (0-12)** |
| **Temporal** | **71(48-138)** | **71(50-138)** | **1 (0-32)** |
| **Maximum** |  |  |  |
| **Highest Single Point** | **213(144-403)** | **236(183-476)** | **5 (0-43)** |
| **Superior** | **200(133-399)** | **203(156-390)** | **5 (0-42)** |
| **Nasal** | **135(75-225)** | **151(76-225)** | **5 (0-51)** |
| **Inferior** | **195(87-403)** | **215(6-476)** | **3 (0-114)** |
| **Temporal** | **118(71-275)** | **116(6-267)** | **3 (0-21)** |
